# 

**Published:** 1874-01-15

**Authors:** 


### *NEW BOOKS RECEIVED.*

The Principles and Practice of Medical Jurisprudence. By Alfred Swaine Taylor, M.D., F.R.S. Second edition. Two vols. Philadelphia: H. C. Lea.

A System of Midwifery; Including the Diseases of Pregnancy and the Puerperal State. By Wm. Leishman, M.D. Philadelphia: H. C. Lea.

Theory and Practice of Medicine. By Fred. T. Roberts, M.D. etc. Philadelphia: Lindsay & Blakiston.

On the Mechanical Treatment of Diseases of the Hip-Joint. By Chas. F. Taylor, New York: Wm. Wood & Co.

